# Maternal Hyperleptinemia Is Associated with Male Offspring’s Altered Vascular Function and Structure in Mice

**DOI:** 10.1371/journal.pone.0155377

**Published:** 2016-05-17

**Authors:** Kathleen A. Pennington, Francisco I. Ramirez-Perez, Kelly E. Pollock, Omonseigho O. Talton, Christopher A. Foote, Constantino C. Reyes-Aldasoro, Ho-Hsiang Wu, Tieming Ji, Luis A. Martinez-Lemus, Laura C. Schulz

**Affiliations:** 1 Department of Obstetrics, Gynecology, and Women’s Health, University of Missouri, Columbia, Missouri, United States of America; 2 Dalton Cardiovascular Research Center, University of Missouri, Columbia, Missouri, United States of America; 3 Department of Biological Engineering, University of Missouri, Columbia, Missouri, United States of America; 4 Division of Biological Sciences, University of Missouri, Columbia, Missouri, United States of America; 5 School of Engineering and Mathematical Sciences, City University London, London, United Kingdom; 6 Department of Statistics, University of Missouri, Columbia, Missouri, United States of America; 7 Department of Medical Pharmacology and Physiology, University of Missouri, Columbia, Missouri, United States of America; Augusta University, UNITED STATES

## Abstract

Children of mothers with gestational diabetes have greater risk of developing hypertension but little is known about the mechanisms by which this occurs. The objective of this study was to test the hypothesis that high maternal concentrations of leptin during pregnancy, which are present in mothers with gestational diabetes and/or obesity, alter blood pressure, vascular structure and vascular function in offspring. Wildtype (WT) offspring of hyperleptinemic, normoglycemic, Lepr^db/+^ dams were compared to genotype matched offspring of WT-control dams. Vascular function was assessed in male offspring at 6, and at 31 weeks of age after half the offspring had been fed a high fat, high sucrose diet (HFD) for 6 weeks. Blood pressure was increased by HFD but not affected by maternal hyperleptinemia. On a standard diet, offspring of hyperleptinemic dams had outwardly remodeled mesenteric arteries and an enhanced vasodilatory response to insulin. In offspring of WT but not Lepr^db/+^ dams, HFD induced vessel hypertrophy and enhanced vasodilatory responses to acetylcholine, while HFD reduced insulin responsiveness in offspring of hyperleptinemic dams. Offspring of hyperleptinemic dams had stiffer arteries regardless of diet. Therefore, while maternal hyperleptinemia was largely beneficial to offspring vascular health under a standard diet, it had detrimental effects in offspring fed HFD. These results suggest that circulating maternal leptin concentrations may interact with other factors in the pre- and post -natal environments to contribute to altered vascular function in offspring of diabetic pregnancies.

## Introduction

Exposure to either gestational diabetes mellitus (GDM) or maternal obesity during prenatal development impacts cardiometabolic health of offspring. Programming of offspring metabolism has been particularly well studied, [1, 2] and although not as abundant, there is also increasing evidence of hypertension in offspring of obese and diabetic pregnancies. For example, a meta-analysis of thirteen cohort studies found that boys born to GDM mothers have elevated systolic blood pressure [3]. This is also seen in animal models, where offspring of rats with streptozotocin-induced type I diabetes have significantly higher blood pressures than offspring of control rats [4–6]. Similarly, both male and female offspring of mice with diet-induced obesity and insulin resistance are hypertensive [7, 8].

Despite the above evidence, little is known about the mechanisms by which GDM and maternal obesity lead to hypertension in offspring. One potential mechanism involves presence of elevated neonatal leptin concentrations in offspring of obese mothers with GDM. Kirk and colleagues found that in progeny of obese, insulin resistant dams, offspring hypertension was associated with neonatal hyperleptinemia [9], and the programming effect of offspring hypertension could be partially recapitulated by injecting offspring of control dams with leptin from postnatal days 9–15 [10]. However, it is not known whether maternal hyperleptinemia in the prenatal period also influences the development of hypertension in offspring.

Leptin is an adipokine that regulates energy homeostasis [11], and directly impacts vascular function in adulthood [12–14]. Pregnancies complicated by obesity and GDM are associated with maternal leptin resistance accompanied by hyperleptinemia [15–19]. Thus, we hypothesize that prenatal exposure to maternal hyperleptinemia may cause vascular dysfunction and hypertension in offspring. Alternatively, the problem in GDM pregnancies may be leptin resistance, i.e. the absence of leptin signaling in the mother, in which case raising maternal leptin would be predicted to protect offspring [20].

To distinguish between these possibilities (and the null hypothesis, that maternal hyperleptinemia does not affect offspring vascular function positively or negatively), we have utilized a genetic model of maternal hyperleptinemia, the Lepr^db/+^ mouse. Under some conditions [20, 21], but not others [22], Lepr^db/+^ mice develop gestational diabetes, and their offspring become fatter and glucose intolerant [23, 24]. In our laboratory conditions, on the C57Bl/6 background, Lepr^db/+^ dams are profoundly hyperleptinemic, and slightly heavier than controls, but have no impairment in glucose tolerance [25]. Hyperleptinemia in this Lepr^db/+^ model has the same effect on offspring metabolism as hyperleptinemia induced by delivering exogenous leptin [25, 26]. Specifically, offspring of non-diabetic Lepr^db/+^ dams and of leptin-infused dams weigh less, are more active, more sensitive to insulin, and have less hepatic triglyceride accumulation than offspring of control dams [25–27], showing that prenatal exposure to maternal hyperleptinemia is beneficial to offspring’s metabolism. Here, we use the same offspring of control and non-diabetic Lepr^db/+^ dams to test the effects of maternal hyperleptinemia on offspring hypertension, in the absence of maternal diabetes and obesity. Because male offspring of Lepr^db/+^ dams exhibited the largest and most consistent differences in body weight and insulin sensitivity [25, 28], these were used to examine effects on hypertension and vascular function. This model has the additional advantage of avoiding any stress caused by treatment with exogenous leptin.

To uncover the mechanisms by which maternal hyperleptinemia may alter offspring risk of hypertension, we examined resistance artery structure and function, as there is evidence of endothelial dysfunction in cells collected from newborns following delivery to a GDM pregnancy [29–32]. In addition, offspring from rodent models of maternal type I and II diabetes have impaired mesenteric artery vasodilatory responses to acetylcholine and bradykinin, but normal responses to nitric oxide (NO), indicative of endothelial dysfunction [4–7].

Resistance arteries play a major role in the regulation of blood pressure, and alterations in resistance artery vasodilation and vasoconstriction are often associated with vascular remodeling processes that modify the passive internal diameter and wall-cross sectional area (CSA) of blood vessels. In due course, these changes in vascular function and structure are important contributors to cardiovascular disease (CVD) [33]. Resistance artery remodeling encompasses extensive and dynamic structural changes in cytoskeletal organization, cell-to-cell connections and extracellular matrix interactions that are controlled by a myriad of mechanical and neurohumoral stimuli [33–35]. In particular, inward eutrophic remodeling, defined as a reduced passive luminal diameter and increased media/lumen ratio without changes in CSA of the vascular wall, is the most common structural change observed in resistance arteries of individuals suffering from essential hypertension, and its presence is highly predictive of life threatening cardiovascular events.[36–38]. Thus, to determine how maternal hyperleptinemia without insulin resistance or obesity affects the risk of hypertension in offspring, blood pressure measurements and detailed analyses of resistance artery reactivity and structural remodeling were conducted in male offspring of wild type (WT)-control and Lepr^db/+^ dams, at 6 weeks of age (juvenile) and at 31 weeks of age (adult) after challenging offspring with a high fat, high sugar diet.

## Materials and Methods

### Animals and Tissue Collection

All animal procedures were approved by the University of Missouri–Columbia Institutional Animal Care and Use Committee and performed in accordance with the National Institutes of Health Guide for the Care and Use of Laboratory Animals. Animals were housed in a 12/12 hour light/dark cycle at 20°C to 26°C with 30% to 70% humidity. Lepr^db/+^ male mice (Strain: B6.BKS(D)-Lepr^db^/J; stock number: 000697) obtained from Jackson Laboratory (Bar Harbor, Maine) were mated to C57Bl/6 WT females bred at the University of Missouri to establish the Lepr^db^ colony. WT females came either from this colony, or directly from the Jackson Laboratory.

**Fig 1:**
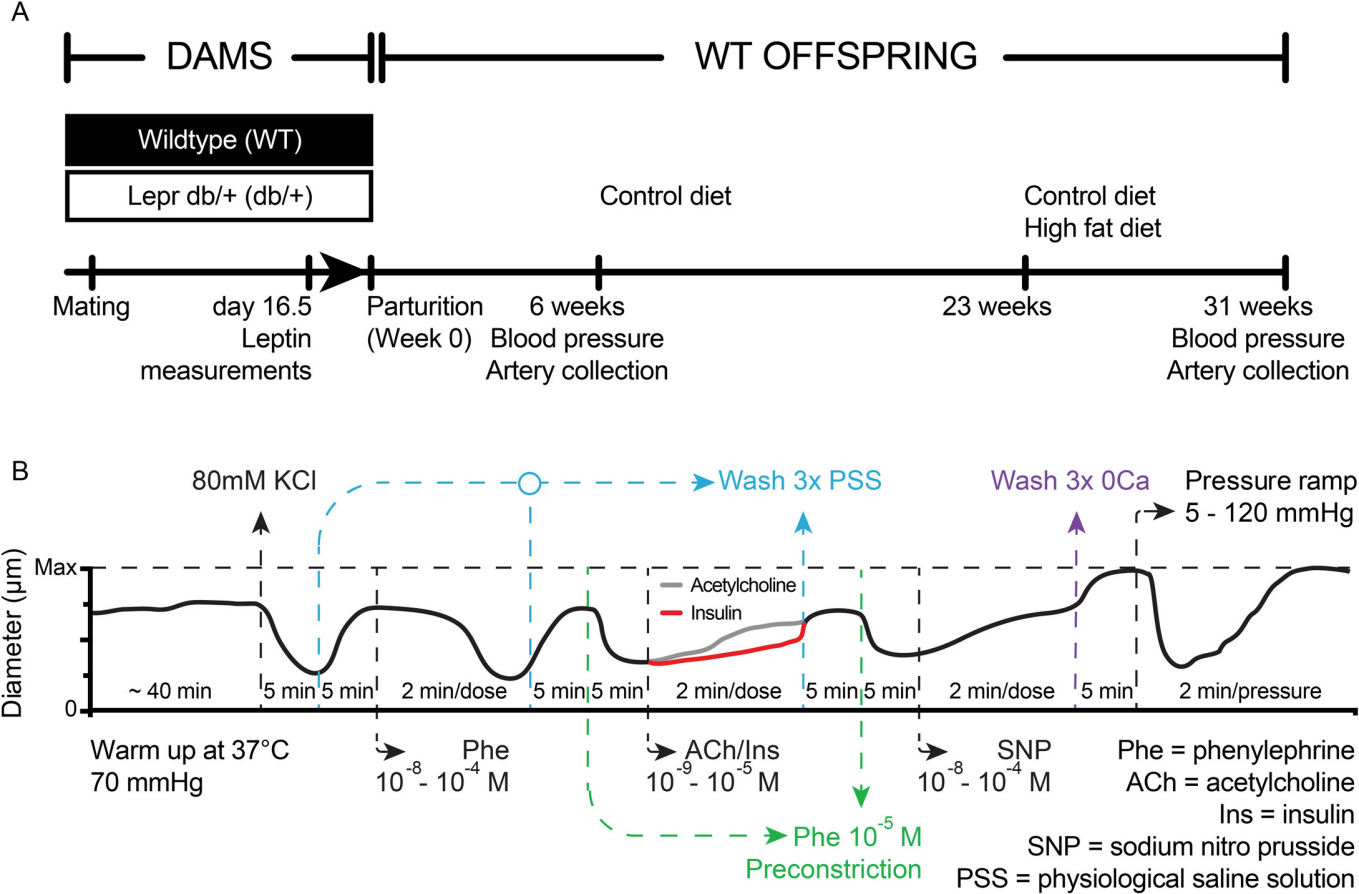
Experimental Design. (A) Animal experiments. Blood pressure (BP) and mesenteric artery structure and function were examined in male wild type (WT) offspring of WT-control and hyperleptinemic Lepr^db/+^ dams. (B) Protocol to test vascular reactivity in isolated, cannulated and pressurized mesenteric resistance arteries. Two arteries were tested for each mouse. The red and green lines indicate that only one of the arteries from each mouse was exposed to either insulin or acetylcholine. At the end of each experiment all arteries were incubated in calcium-free buffer to obtain maximal passive diameters and subsequently exposed to varying levels of intraluminal pressure.

An overview of the experiments involving animals is presented in Fig 1A. In order to maintain the same genetic heterogeneity within litters, WT females were mated to leptin receptor heterozygous knockout (Lepr^db/+^) males and Lepr^db/+^ females were mated to WT males and only WT offspring of each cross were followed. As previously reported, on day 16.5 of pregnancy, the Lepr^db/+^ dams were hyperleptinemic based on fasting leptin levels compared to the WT control mothers [25]. Litter sizes were not affected by maternal hyperleptinemia [25]. Weights, behaviors and metabolic characteristics have been reported previously for the mice used in this study [25, 28]. For this study, juvenile (6 week old) and adult (31 week old) WT male offspring were evaluated for alterations in blood pressure as described below. Two WT male offspring from each mother were followed for 31 weeks. At 23 weeks of age, when offspring were fully adult, half were placed on a high fat, high sugar diet (HFD, 45% kcal/fat, 17% kcal/sucrose, Research Diets D12451) to identify any fetal programming effects that might interact with offspring diet. This period was chosen to permit comparison to a previous study of maternal hyperleptinemia with food restriction [39]. Following arterial blood pressure measurements at 6 or 31 weeks of age, all mice were euthanized by inducing pneumothorax followed by exsanguination, while under anesthesia and tissues were harvested for further analysis. Mesenteric arteries were studied as described below. Hearts were fixed in 4% paraformaldehyde (PFA) overnight and then embedded in optimal cutting temperature (O.C.T.) compound (Fisher Scientific, Pittsburgh, PA) for analysis.

### Blood Pressure Analysis

To determine if maternal hyperleptinemia altered offspring blood pressure, systolic and diastolic blood pressures were measured in juvenile (6 week old) and adult male (31 week old) offspring from WT-control and Lepr^db/+^ dams using a tail cuff CODA non-invasive blood pressure system (Kent Scientific, Torrington CT, USA) as well as by carotid catheter under anesthesia as previously described [40]. For tail-cuff measurements, individual animals were placed in a commercially acquired restraining tube and allowed to acclimatize for 10 minutes prior to initiating the blood pressure measurement protocol. For the invasive blood pressure measurements, mice were anesthetized with inhaled isoflurane and while under surgical plane anesthesia at 2% isoflurane, a carotid artery was cannulated and arterial pressure measured using a PowerLab data acquisition system and LabChart software (ADInstruments) as previously described [40].

### Mesenteric Resistance Artery Functional Analyses

Vascular reactivity was evaluated in two second-order mesenteric resistance arteries (186–301 μm in internal diameter) harvested from offspring following blood pressure analysis as previously described [41, 42]. Briefly, to evaluate vasoconstrictor responses, mesenteric artery segments from each mouse were exposed to 80 mM KCl (equimolarly substituted for NaCl) to test for viability and then to cumulative concentrations of phenylephrine to study adrenergic-dependent vasoconstriction. To evaluate endothelium-dependent vasodilation, one of the arteries was exposed to insulin, and the second to acetylcholine (ACh). Finally, both arteries were exposed to cumulative concentrations of sodium nitroprusside (SNP) to evaluate endothelium-independent vasodilatory responses. All vasodilatory responses were assessed on arteries pre-constricted with 10^−5^ M phenylephrine (Fig 1B), as there were no differences in phenylephrine-induced vasoconstriction responses between the experimental groups and all arteries exhibited similar levels of constriction in response to this concentration of phenylephrine.

### Confocal/Multiphoton Microscopy Imaging of Mesenteric Arteries

At the end of each experiment, vessels were fixed in 4% paraformaldehyde, while pressurized at 70 mmHg for 1 hour. For imaging, vessels were rinsed twice in phosphate buffered saline (PBS) and once in 0.1 M Glycine for 5 minutes each time. Cannulated vessels were flushed with 1 mL PBS to rinse their lumen and permeabilized via incubation in 0.5% Triton X-100 for 20 minutes. Vessels were washed twice in PBS and incubated for 1 hour in 0.5 μg/mL 4',6-diamidino-2-phenylindole (DAPI), 0.2 μM Alexa Fluor 633 Hydrazide (Molecular Probes) and 0.02 μM Alexa Fluor 546 phalloidin (Molecular Probes) in PBS. After being washed 3 times in PBS, vessels were imaged using a Leica SP5 confocal/multiphoton microscope with a 63x/1.2 numerical aperture water objective. Alexa Fluor 633, to image elastin, was excited with a 633 nm HeNe laser. Alexa Fluor 546 phalloidin, to image F-actin components, was excited with a 543 nm HeNe laser. DAPI, to image nuclei, was excited with a multi-photon laser at 720 nm. Collagen was imaged via second-harmonic image generation using a multi-photon laser at 850 nM. All imaging and image analyses were performed as previously described [43].

### Elasticity Measurements in Mesenteric Arteries

Circumferential strain, circumferential stress, Young’s modulus of elasticity, and compliance were all calculated using the internal diameter and wall thickness measurements obtained during the pressure-diameter curves generated while vessels were under passive conditions. The number and area of fenestrae in the internal elastic lamina (IEL) were determined and the modulus of elasticity specific for the IEL was calculated using images generated with Alexa 633 staining as previously described [43].

The circumferential strain was calculated as the difference between the intraluminal diameter obtained under passive conditions at each intraluminal pressure level (*Dp*) minus the diameter at the lowest pressure tested (5 mmHg) divided by the diameter at lowest pressure [35]:

$$\epsilon =\frac{D_{P}-D_{5\text{mmHg}}}{D_{5\text{mmHg}}}.$$

Circumferential stress was calculated by using the formula for thin-walled vessels, where *P* is the intraluminal pressure, and *τ* is wall thickness:

$$\sigma =\frac{P*D_{P}}{2\tau },$$

Compliance (C) provides information on arterial wall stiffness. It is particularly sensitive at low pressures and was calculated with the following formula [44], where Δ*A* is the change in the CSA of the wall relative to an increment in intraluminal pressure Δ*P*:

$$C=\frac{\Delta \text{A}}{\Delta \text{P}}.$$

The Young’s modulus of elasticity provides information about arterial stiffness, particularly at high levels of intraluminal pressure [35]. It is the slope in the strain-stress curve at each pressure as represented in the formula below [35].

$$E=\frac{\sigma }{\epsilon }.$$

The elastic modulus can be separated in two portions depending on the level of intravascular pressure in order to separate the moduli dominated by elastin and that dominated by collagen [45–47]. We chose the first three values in the lower rage of intravascular pressures (Region I: 5, 10, 20 mmHg) to provide information on the low Young modulus of elasticity (E_low_). At low pressures elastin dominates the elastic behavior of the artery. At higher pressures the dominant element is collagen and we chose the last three values of intraluminal pressure (Region II: 80, 100, 120 mmHg) to determine the high Young modulus of elasticity (E_high_). E_low_ and E_high_ represent the slope of the linear regressions of Regions I and II respectively.

The modulus of elasticity specific for the internal elastic lamina (IEL) can be interpreted as an array of coupled springs, where the fenestrae in the IEL are vertices between the springs. Under this consideration, the elastic modulus can be associated to an area fraction in the elastic lamina. The area fraction is proportional to the number of holes or fenestrae (*n*) and their area (*A*) (all sizes are the same) [48].

p=1−nA.

The number of holes per unit area is based in the symmetry used in the model. We used a honeycomb symmetry (*n* = 2) paradigm. Percolation ocurrs when the circular holes touch. For a honeycomb array the critical area fraction of material is $p_{c}=1-\pi /3\sqrt{3}=0.395$. The normalized Young's modulus (*φ*) as a function of the percolation of the elastic lamina in the critical domain is:

$$\varphi_{I}\left(p\right)=\frac{E}{E_{0}}\approx \frac{2}{\pi \sqrt{3}}{\left(\frac{p-p_{c}}{1-p_{c}}\right)}^{1/2},$$

While in the diluted limit is:

$$\varphi_{II}\left(p\right)=\frac{E}{E_{0}}=3p-2.$$

The total behavior of *E*/*E*_0_ is described using an interpolation of the two regions *φ*_*I*_ and *φ*_*II*_.

#### Heart Histological Analysis

To determine if cardiac lipid accumulation was affected by maternal hyperleptinemia, histological analysis was completed on adult male offspring hearts using Oil Red O staining based on published guidelines [49]. Frozen hearts were embedded and sectioned at 12 μm and 3 sections at 100 μm intervals were placed on a slide. Oil red O staining was then performed as described [49]. Quantitative analysis was performed as previously described [49] using imageJ software (NIH).

Cardiac fibrosis was also evaluated in male offspring by Picro-Sirius Red staining. Staining was performed on the heart by the histology core at IDEXX BioResearch Radil facility (Columbia, MO, USA) to assess fibrotic lesions. Tissue was visualized with a light microscope.

### Statistical Analyses

All data analyses were performed using Statistical Analysis System software version 9.4 (SAS^®^, SAS institute, Cary, NC). Data are presented as means ± SE. Values of P ≤ 0.05 were considered significant.

For the data analysis of vascular functional responses, before model fitting, samples that did not constrict at least 20% relative to the maximal passive diameter with the application of high KCl were removed from analysis. For each of the applications of vasodilatory agonists (acetylcholine, insulin, and SNP), samples that did not constrict at least 20% relative to the maximal diameter with the initial application of phenylephrine were removed from the analysis of that vasoactive agent.

The offspring arterial diameters were modeled to determine which factors were significant when testing the response of the sample with each of the vasoactive agents: phenylephrine, acetylcholine, insulin, and SNP, respectively. The diameters were normalized to represent values relative to their maximal diameter and initial constriction to account for the large variability amongst the arterial diameters. These normalized values were regressed to the maternal environment (Lepr^db/+^ and WT), offspring diet (HFD-fed and standard diet (SD)-fed), and nine levels of agonist (over time) as well as interaction effects between each two and three factors. Repeated measurements of each artery of an offspring were taken at several time points for each application of acetylcholine and insulin. Mean of two repeated measurements from two arteries of the same offspring were taken at each time point for both applications of phenylephrine and SNP. Thus, to account for the correlation among the repeated measurements, a heterogeneous compound symmetry correlation structure was used. This correlation structure was chosen based on AIC (Akaike information criterion), BIC (Bayesian information criterion), and its biological interpretation. Each model was constructed using backward elimination. The main effects and interaction effects were selected by controlling the statistical significance at level 0.10. The studentized residual plots for each of the models suggest that model assumptions are met in all cases. The Kenward-Roger adjustment to denominator degrees of freedom was used when fitting all three models in this study because of the unbalanced design, random effects, repeated measures, and moderate sample size. After model selection, the Tukey-Kramer HSD (honest significant difference) method was used to adjust multiple tests in comparing different levels of treatment combinations.

The structural and elastic properties of mesenteric arteries were modeled to determine which factors (maternal environment, Lepr^db/+^ or WT), offspring diet (HFD-fed or SD-fed), pressure, and interactions were significant. The same model construction and selection procedures described in the data analysis for the vascular functional responses were utilized for data analysis here. Residual plots were examined to ensure model assumptions are met. Similarly, the Kenward-Roger degree of freedom was used since experiments are of unbalanced design with correlated observations. Finally, Tukey-Karmer HSD was used to adjust multiple tests.

## Results

### Maternal hyperleptinemia did not alter offspring blood pressure

**Fig 2:**
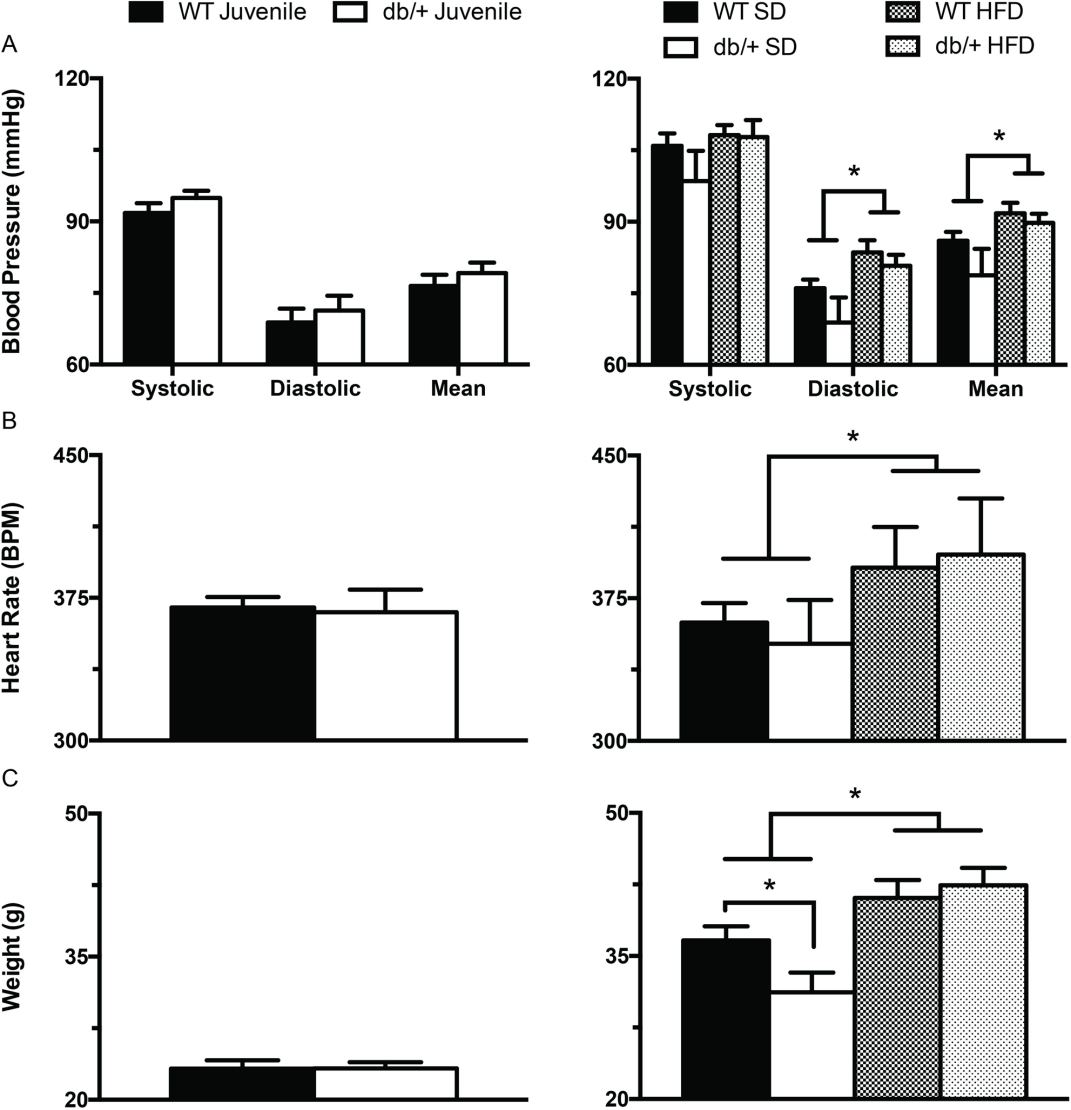
Effect of maternal environment and offspring diet on blood pressure (obtained via carotid catheter), heart rate and body weight of juvenile (6 week-old) and adult (31 week-old) wild type male offspring. (A) Systolic, diastolic and mean arterial pressures in 6 (left panel) and 31 (right panel) week old offspring. (B) Heart rate in 6 (left panel) and 31 (right panel) week old offspring. (C) Body weights in 6 (left panel) and 31 (right panel) week old offspring. Data are means ± SEM of n = 5–7 number of animals per treatment group combination. *P<0.05. WT, wild type; db/+, Lepr^db/+^; SD, standard diet; HFD, high fat diet.

To determine the effects of maternal hyperleptinemia on offspring hypertension, blood pressures were measured in both conscious and anesthetized animals. Blood pressure and heart rate were obtained at either 6 or at 31 weeks of age (Fig 1) in offspring fed SD or HFD. There were no significant differences in blood pressure or heart rate in juvenile or adult offspring when measured in conscious animals using the tail-cuff method (data not shown). In adult offspring, catheter measurements of diastolic blood pressure, mean arterial pressure, and heart rate were significantly increased (*P*<0.05) by consumption of HFD, regardless of prenatal exposure to hyperleptinemia (Fig 2).

The increases in blood pressure and heart rate that occurred with HFD-feeding paralleled an increase in body weight (Fig 2C). The adult male offspring from Lepr^db/+^ dams weighed less at sacrifice than offspring from WT dams only when the offspring were fed SD (Fig 2C). However, these mice were a subset of those used in a previous analysis, and when the larger group was examined, offspring of Lepr^db/+^ dams weighed less regardless of offspring diet [25]. These differences in offspring weight associated with maternal hyperleptinemia were not matched by differences in offspring blood pressure or heart rate (Fig 2).

### Responses to ACh and insulin were affected differentially by maternal hyperleptinemia, depending on offspring diet

**Fig 3:**
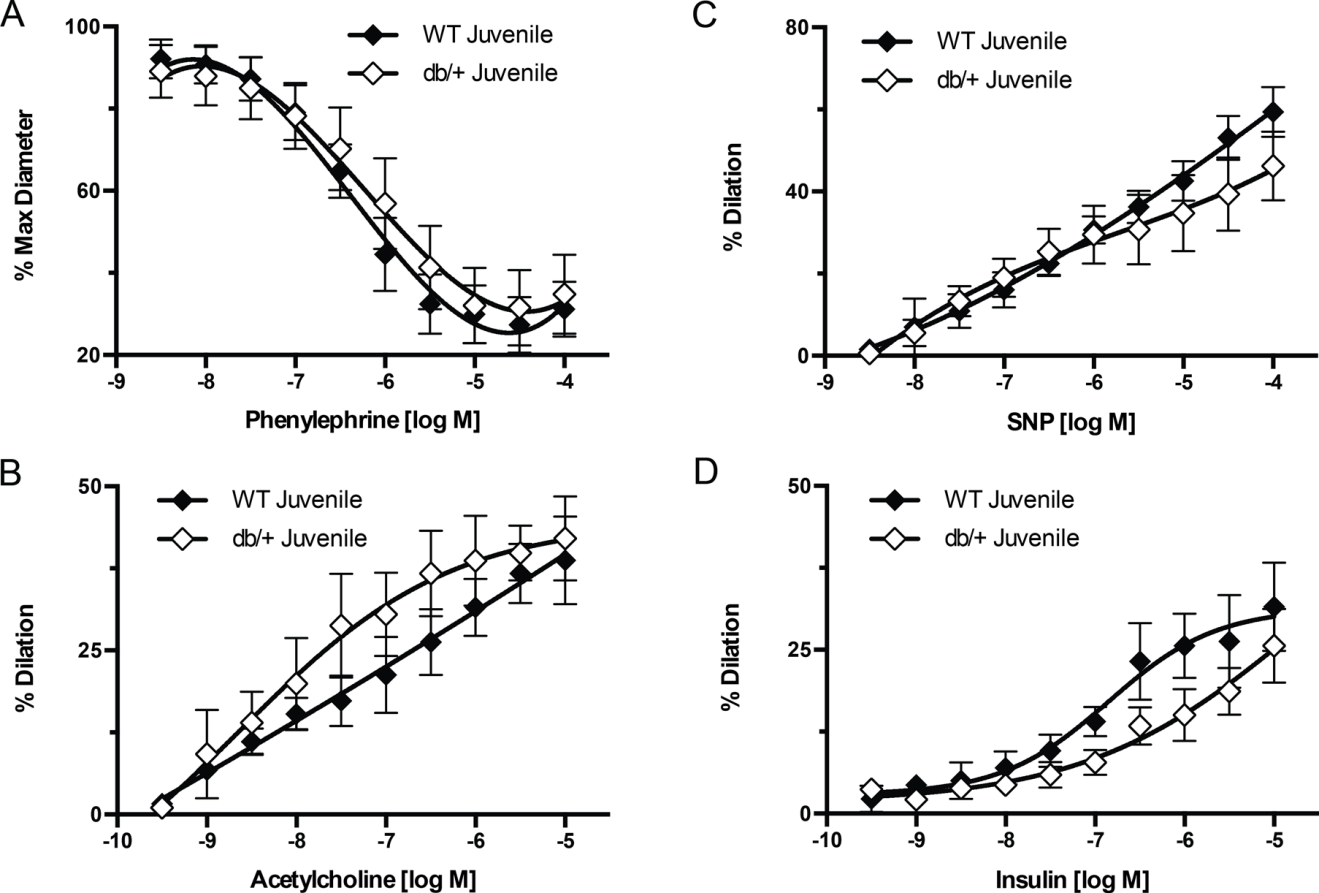
Effect of maternal environment on mesenteric artery responses to vasoactive agonists. Mesenteric arteries were obtained from juvenile (6 week-old) wild type (WT) male offspring of WT-control or Lepr^db/+^ (db/+) dams. (A) Vascular responses to increasing concentrations of phenylephrine. (B) Vascular responses to increasing concentrations of acetylcholine. (C) Vascular responses to increasing concentrations of sodium nitroprusside (SNP). (D) Vascular responses to increasing concentrations of insulin. Data are means ± SEM of n = 4–5 number of animals (vessels) per treatment group combination.

We further examined the effect of maternal hyperleptinemia on offspring vascular function by examining mesenteric resistance artery vasoconstriction and vasodilation responses in the male WT offspring of WT-control and hyperleptinemic Lepr^db/+^ dams. Mesenteric artery vasodilation and vasoconstriction responses in juvenile offspring maintained on a SD were not affected by maternal environment (Fig 3).

**Fig 4:**
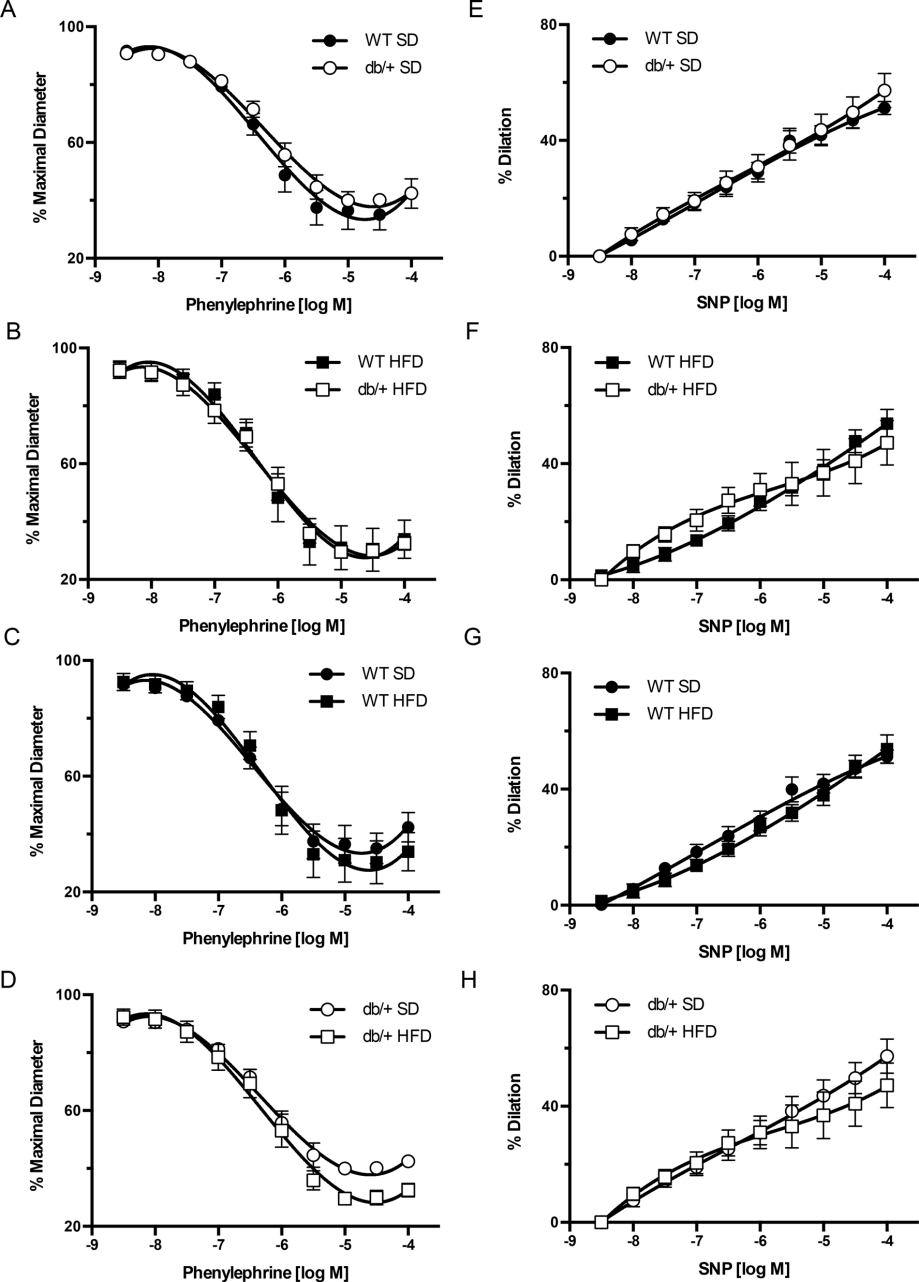
Effect of maternal environment and offspring diet on mesenteric resistance artery responses to phenylephrine and sodium nitroprusside (SNP). All blood vessels were obtained from adult (31 week-old) wild type (WT) male offspring of WT-control or Lepr^db/+^ (db/+) dams. (A-D) Phenylephrine-induced vasoconstriction responses. (E-H) SNP-induced vasodilatory responses. Data are means ± SEM of n = 5–7 number of animals (vessels) per treatment group combination. SD, standard diet; HFD, high fat diet.

The interacting effects of (maternal environment) x (offspring diet) on vascular function were evaluated in mesenteric arteries from adult offspring. Vasoconstriction in response to alpha-1 adrenergic stimulation was tested using phenylephrine. Arterial vasoconstriction responses to incremental concentrations of phenylephrine did not differ between offspring from WT and Lepr^db/+^ dams, regardless of offspring diet (Fig 4A–4D). Endothelial-independent vasodilation was also tested, and no significant differences were observed for SNP-induced vasodilation among any of the groups (Fig 4E–4H).

**Fig 5:**
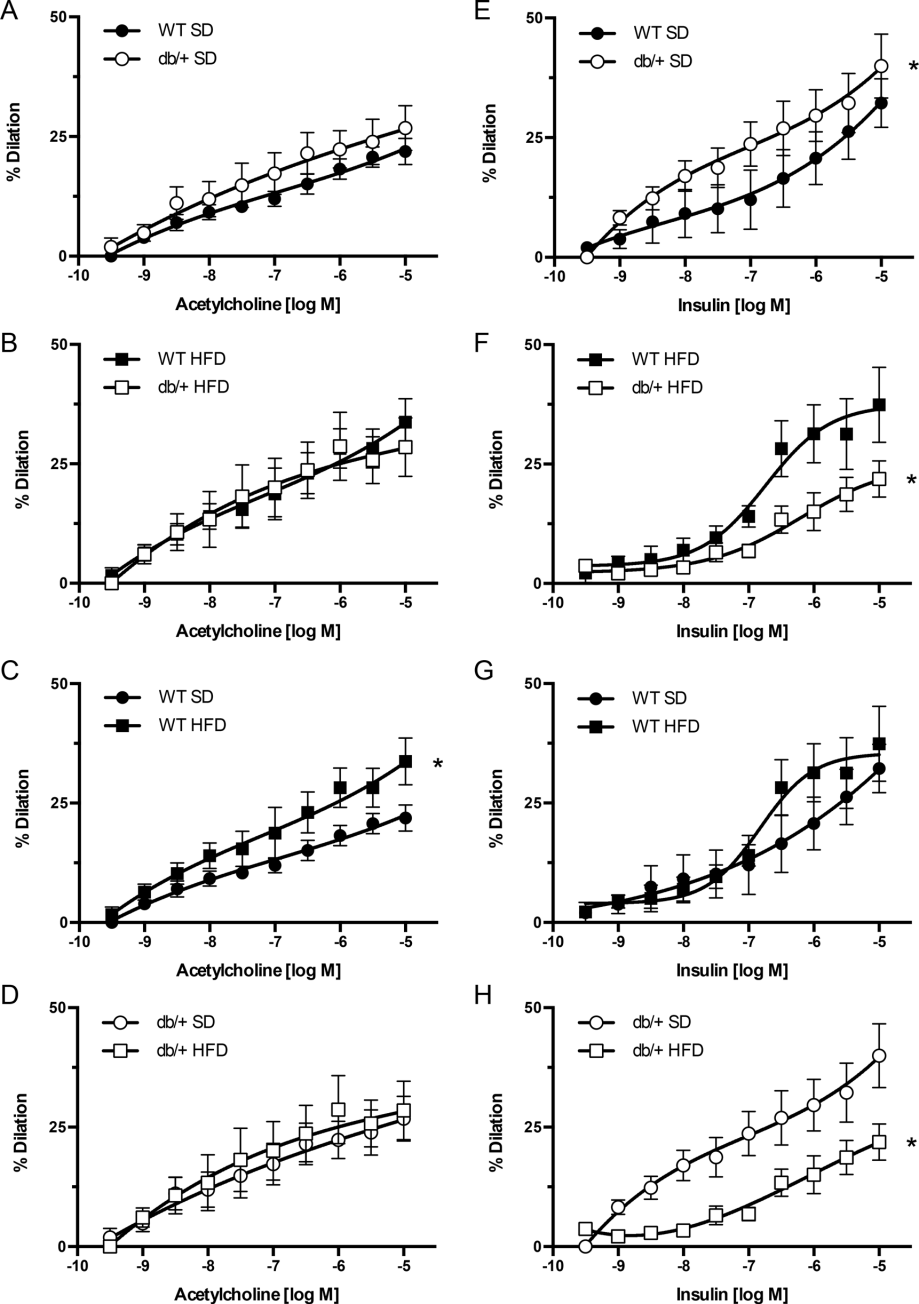
Effect of maternal environment and offspring diet on mesenteric resistance artery responses to acetylcholine and insulin. All blood vessels were obtained from adult (31 week-old) wild type (WT) male offspring of WT-control or Lepr^db/+^ (db/+) dams. (A-D) Acetylcholine-induced vasodilatory responses. (E-H) Insulin-induced vasodilatory responses. Data are means ± SEM of n = 5–6 number of animals (vessels) per treatment group combination. *P<0.05. SD, standard diet; HFD, high fat diet.

Endothelial-dependent vasodilation responses of mesenteric arteries to ACh (Fig 5A–5D) and insulin (Fig 5E–5H) were also assessed. When offspring were fed a SD, those born to WT and Lepr^db/+^ dams exhibited no differences in ACh-induced vasodilation (Fig 5A). However, offspring of WT dams had greater (*P*<0.05) vasodilatory responses to ACh when fed a HFD than a SD (Fig 5C). No differences in ACh vasodilation responses were observed between arteries of offspring fed the HFD from WT and Lepr^db/+^ dams (Fig 5B) or between SD- and HFD-fed offspring from Lepr^db/+^ dams (Fig 5D).

In offspring fed a SD, the vasodilation response to insulin was significantly greater (*P*<0.05) in arteries from offspring of Lepr^db/+^ dams than in offspring of WT dams (Fig 5E). In contrast, insulin dependent vasodilation was significantly blunted (*P*<0.05) in HFD-fed offspring of Lepr^db/+^ dams compared to HFD-fed offspring of WT-control dams (Fig 5F) and SD-fed offspring of Lepr^db/+^ dams (Fig 5H). There were no differences in insulin-induced vasodilation between SD- and HFD-fed offspring from WT-control dams (Fig 5G).

The elastic properties of mesenteric arteries were affected by a (maternal environment) x (offspring diet) interaction.

**Fig 6:**
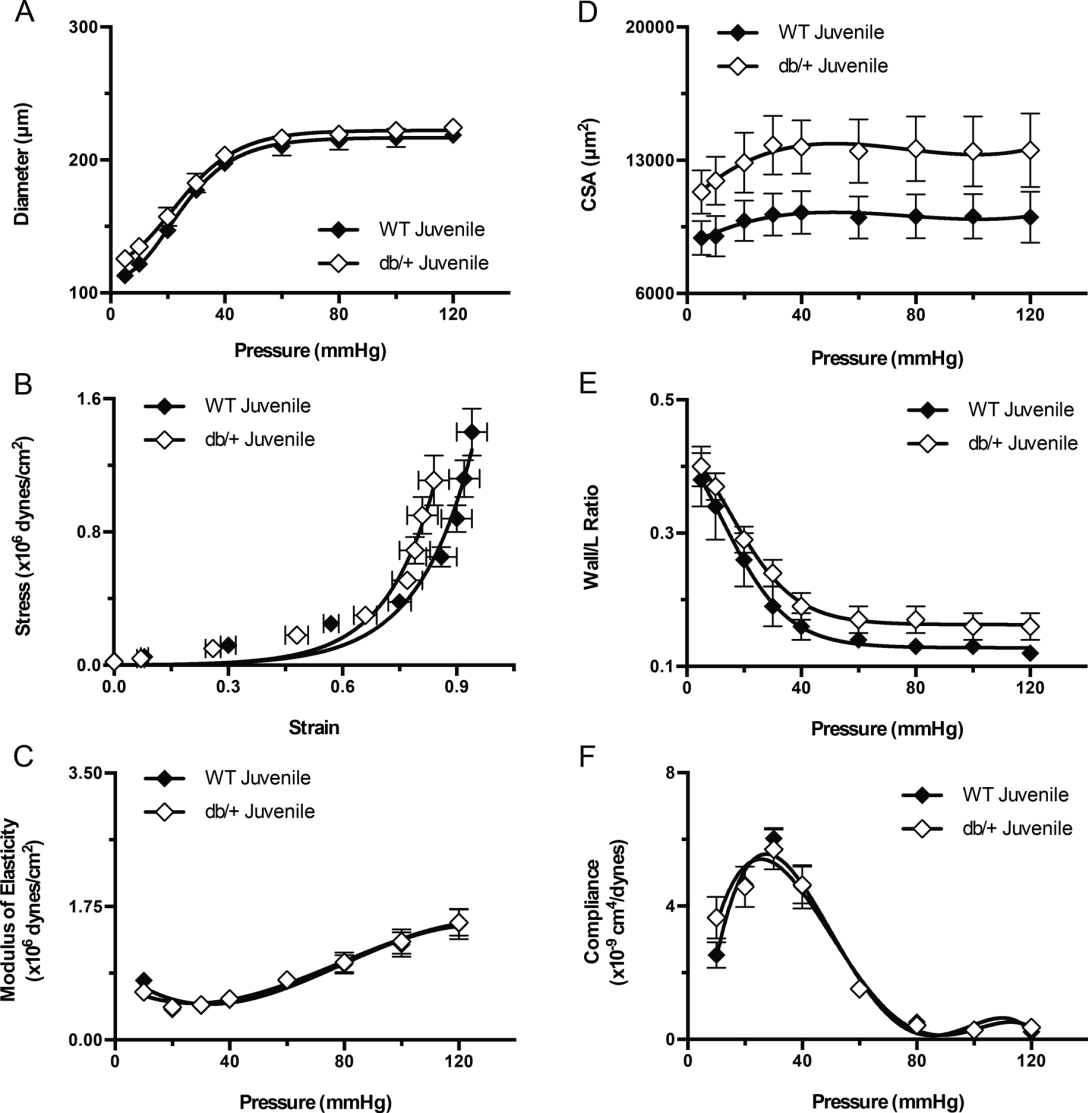
Effect of maternal environment on the structure and mechanical properties of mesenteric resistance arteries from juvenile (6 week-old) wild type (WT) male offspring of WT-control or Lepr^db/+^ (db/+) dams. (A) Pressure-diameter curves of blood vessels kept under passive conditions. (B) Strain-stress relationship curves of mesenteric arteries kept under passive conditions at different intravascular pressures. (C) Elastic moduli of mesenteric arteries kept under passive conditions at different intravascular pressures. (D) Cross sectional area (CSA) of the vascular wall in mesenteric arteries kept under passive conditions at different intravascular pressures. (E) Vascular wall to intravascular lumen ratio of mesenteric arteries kept under passive conditions at different intravascular pressures. (F) Compliance of mesenteric arteries kept under passive conditions at different intravascular pressures. Data are means ± SEM of n = 5 number of animals (vessels) per treatment group combination.

We further examined the structural properties of mesenteric arteries to determine if the observed differences in vasodilation were associated with differences in vascular remodeling. No differences in vascular structural characteristics were observed in juvenile offspring (Fig 6).

**Fig 7:**
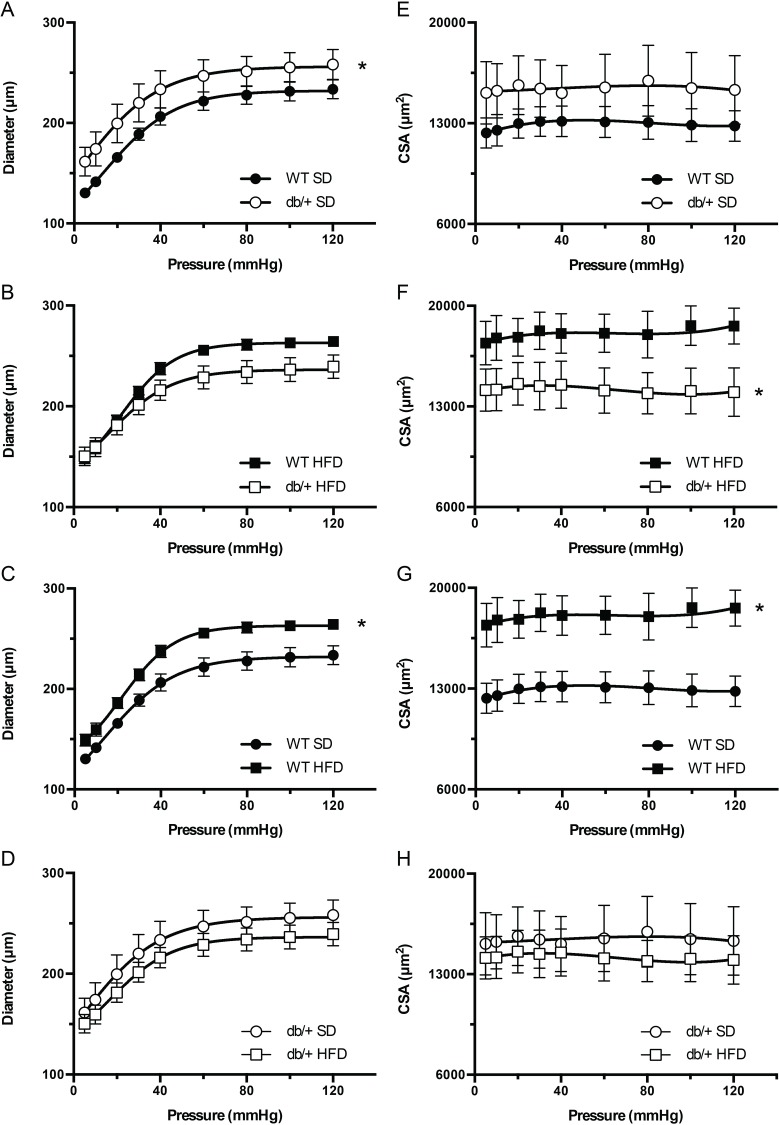
Effect of maternal environment and offspring diet on the structural properties of mesenteric resistance arteries from adult (31 week-old) wild type (WT) male offspring of WT-control or Lepr^db/+^ (db/+) dams. (A-D) Pressure-diameter curves of blood vessels kept under passive conditions. (E-H) Cross sectional area (CSA) of the vascular wall in mesenteric arteries kept under passive conditions at different intravascular pressures. Data are means ± SEM of n = 5–7 number of animals (vessels) per treatment group combination. *P<0.05. SD, standard diet; HFD, high fat diet.

In adult mice, mesenteric vascular remodeling in response to HFD differed between WT male offspring from Lepr^db/+^ and WT-control dams. In offspring fed a SD, the passive luminal diameter of arteries was significantly greater (*P*<0.05) in offspring of Lepr^db/+^ dams than in offspring from WT dams (Fig 7A). HFD feeding significantly increased passive luminal diameters and CSAs in offspring from WT dams (Fig 7C and 7G), but not in offspring from Lepr^db/+^ dams (Fig 7D and 7H). As a result, on HFD, the CSA was significantly reduced (*P*<0.05) in offspring of Lepr^db/+^ dams versus that from WT dams (Fig 7F). No differences were observed in the arterial wall-to-lumen ratios among any of the groups (data not shown).

**Fig 8:**
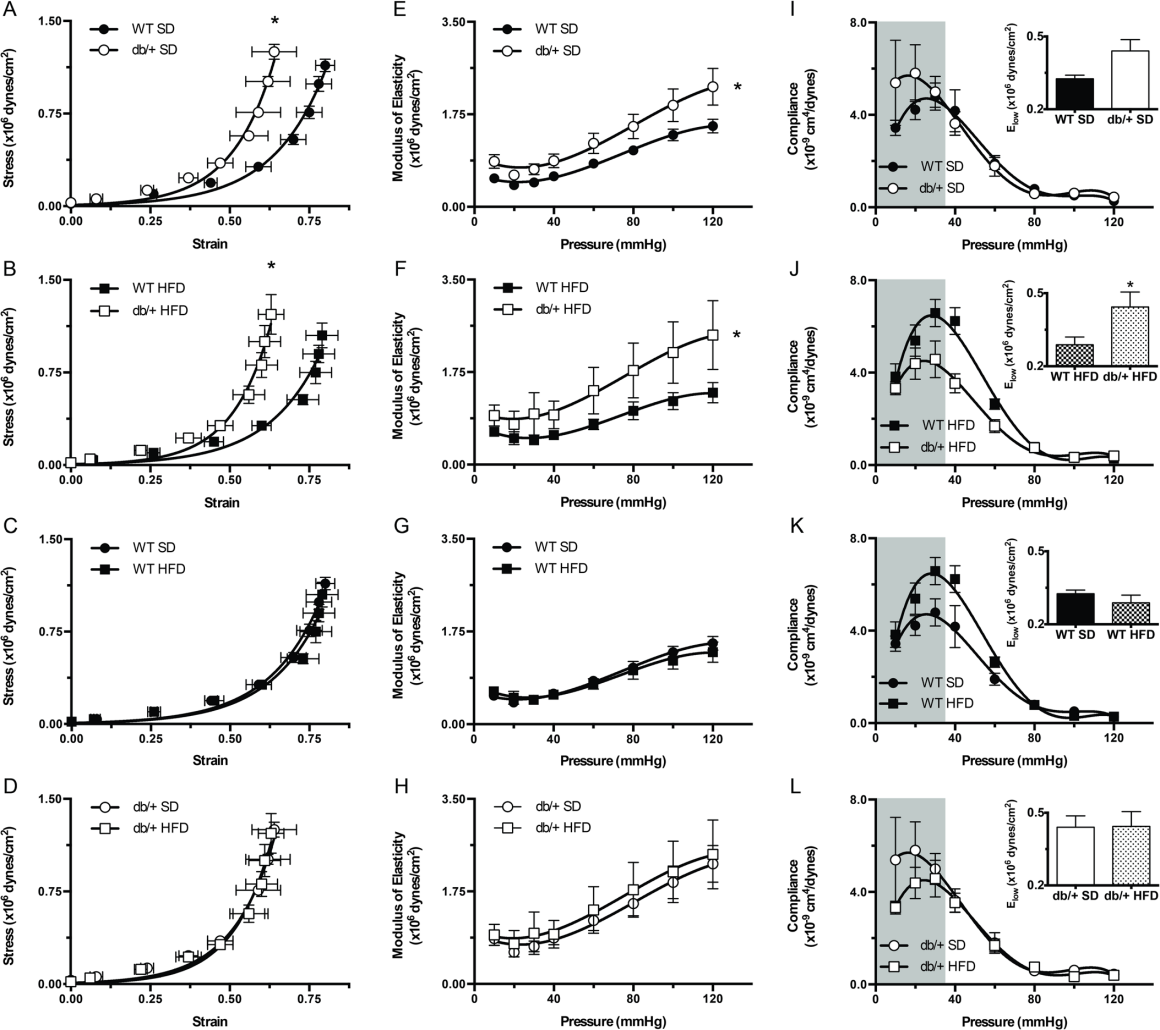
Effect of maternal environment and offspring diet on the mechanical properties of mesenteric resistance arteries from adult (31 week-old) wild type (WT) male offspring of WT-control or Lepr^db/+^ (db/+) dams. (A-D) Strain-stress relationship curves of mesenteric arteries kept under passive conditions at different intravascular pressures. (E-H) Elastic moduli of mesenteric arteries kept under passive conditions at different intravascular pressures. (I-L) Compliance of mesenteric arteries kept under passive conditions at different intravascular pressures. The shaded areas represent the data used to calculate the elastic moduli at low pressures, which are shown in the insets. Data are means ± SEM of n = 5–7 number of animals (vessels) per treatment group combination. *P<0.05. SD, standard diet; HFD, high fat diet.

Arterial stiffness was affected by maternal environment, and diet had an effect depending on maternal environment. Offspring of Lepr^db/+^ dams had significantly reduced (*P*<0.05) vascular wall strain values (Fig 8A and 8B) and higher moduli of elasticity (Fig 8E and 8F), indicative of arterial stiffness compared to offspring of WT dams. There were no differences in vascular wall stress (Fig 8C and 8D) or elastic moduli (Fig 8G and 8H) between diets. To investigate arterial stiffness at low pressures, we analyzed arterial compliance (Fig 8I–8L) and the low-modulus of elasticity (Fig 8I–8L, insets). The low-modulus of elasticity in mesenteric arteries from offspring of Lepr^db/+^ dams was increased (*P*<0.05) compared to offspring of WT dams, when offspring were fed a HFD (Fig 8J, inset). This effect of HFD was correlated with a tendency for an increased vascular compliance at low pressures (Fig 8J); however, the difference in compliance did not reach statistical significance. No other significant effects of maternal environment or diet on arterial compliance or the low-modulus of elasticity were observed. Thus, maternal hyperleptinemia increased arterial stiffness at high pressures, independent of offspring diet, whereas at low pressures, there was a (maternal environment) x (offspring diet) interaction, such that offspring of WT, but not Lepr^db/+^ dams, had reduced arterial stiffness (increased compliance) in response to HFD.

### The structural composition of mesenteric resistance arteries was affected by a (maternal environment) x (offspring diet) interaction

**Fig 9:**
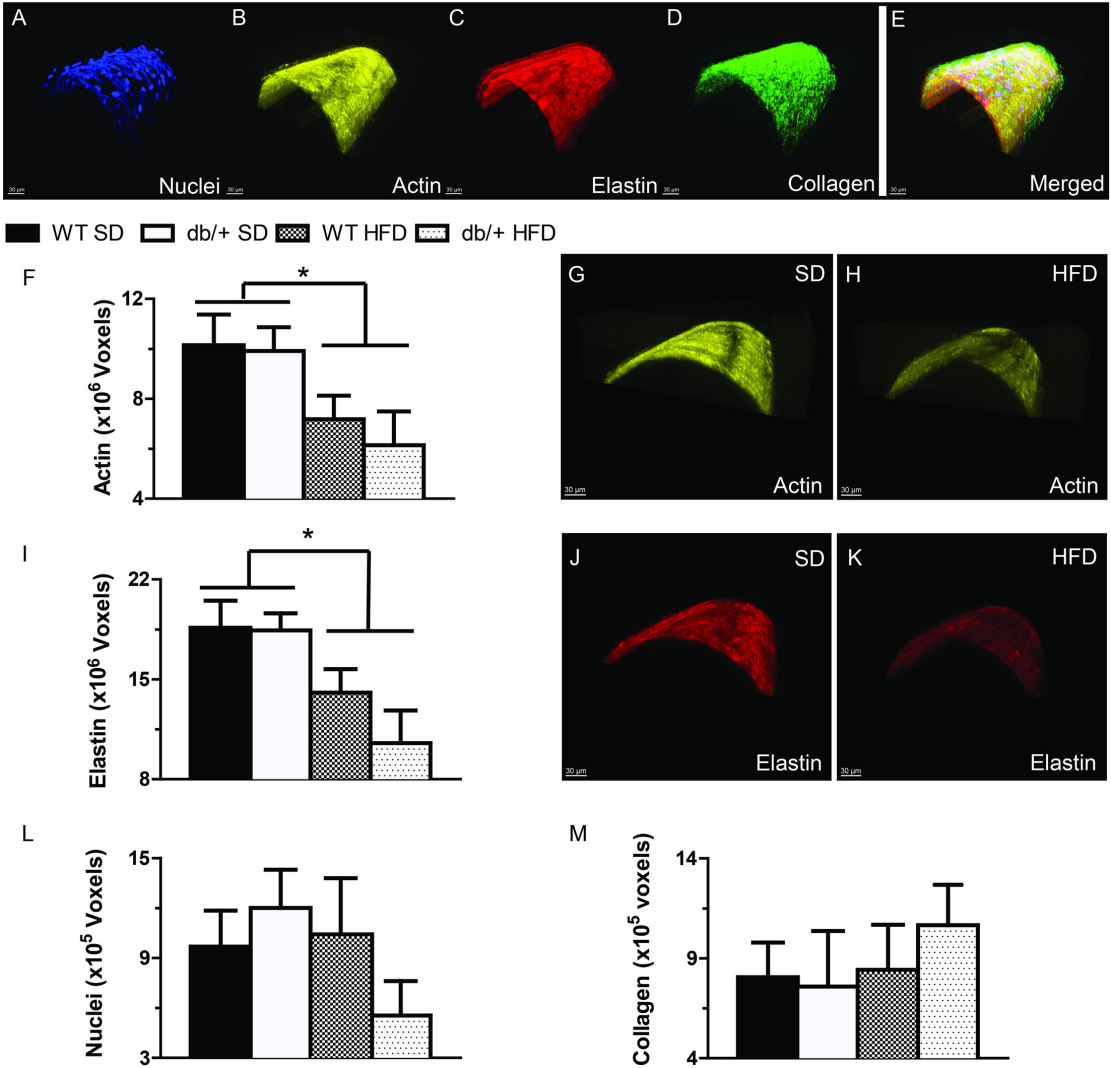
Effect of maternal environment and offspring diet on the morphological characteristics of mesenteric resistance arteries from adult (31 week-old) wild type (WT) male offspring of WT-control or Lepr^db/+^ (db/+) dams. (A-E) Representative confocal images of mesenteric resistance arteries showing (A) nuclei; (B) F-actin; (C) elastin; (D) collagen; and (E) merged image. (F-H) Group data and representative images showing that feeding a high-fat diet was associated with a significant reduction in arterial F-actin content. (I-K) Group data and representative images showing that feeding a high-fat diet was associated with a significant reduction in arterial elastin content. (L) Vascular smooth muscle cell number, represented by nuclei contained within the medial layer of mesenteric arteries. (M) The amount of collagen contained in the wall of mesenteric arteries. Data are means ± SEM of n = 5–7 number of animals (vessels) per treatment group combination. *P<0.05. SD, standard diet; HFD, high fat diet.

The cytoskeletal and extracellular matrix composition of blood vessels directly impacts vascular structure and stiffness. Therefore, we examined F-actin, elastin and collagen contents of mesenteric arteries from adult offspring of WT-control and Lepr^db/+^ dams by confocal/multiphoton microscopy (Fig 9). Maternal environment did not affect the F-actin cytoskeletal composition of these arteries (Fig 9F). However, offspring fed a HFD, regardless of maternal genotype, had significantly reduced (*P*<0.05) F-actin cytoskeletal volume in the medial layer compared to vessels from offspring on a SD (Fig 9F–9H). The elastin content (Fig 9I–9K) was also significantly reduced (*P*<0.05) in HFD-fed offspring compared to SD, regardless of maternal environment. The number of vascular smooth muscle cells and collagen content of the arteries was not different among any of the groups (Fig 9L and 9M).

**Fig 10:**
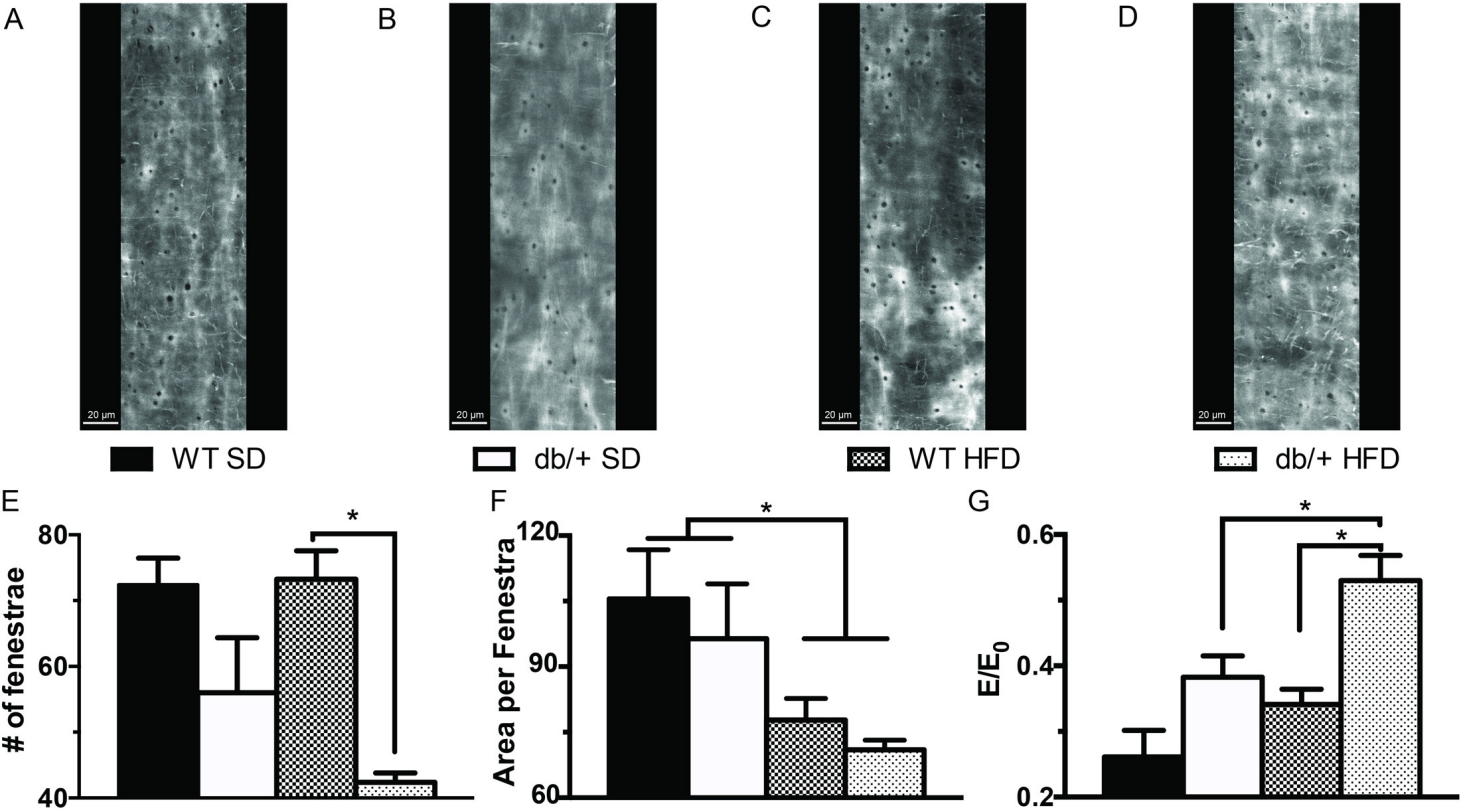
Effect of maternal environment and offspring diet on the internal elastic lamina characteristics of mesenteric resistance arteries from adult (31 week-old) wild type (WT) male offspring of WT-control or Lepr^db/+^ (db/+) dams. (A-D) Representative confocal images of the internal elastic lamina in mesenteric resistance arteries from each of the treatment group combinations. (E-G) Group data showing the number and area of fenestrae within the internal elastic lamina and the elastic modulus of elasticity normalized as a function of the percolation of the internal elastic lamina and its fenestrae. Data are means ± SEM of n = 4–5 number of animals (vessels) per treatment group combination. *P<0.05. SD, standard diet; HFD, high fat diet.

The number and area of fenestrae in the internal elastic lamina (Fig 10) were also evaluated in the mesenteric arteries of the adult offspring. Overall, offspring from Lepr^db/+^ dams had fewer (*P*<0.05) fenestrae compared to offspring from WT dams, regardless of diet, although in pairwise comparisons, the difference was only significant in HFD-fed offspring (Fig 10E). The mean area occupied by each fenestra was also significantly reduced by HFD (Fig 10F). The calculation of the internal elastic lamina elastic moduli normalized by the fenestrae critical value (E/E0) was significantly affected by maternal environment and diet. The E/E0 was significantly greater (*P*<0.05) in vessels from mice fed a HFD and in vessels from offspring of Lepr^db/+^ dams (Fig 10G).

### Cardiac lipid accumulation and fibrosis were not affected by maternal environment or offspring diet

Lipid accumulation and fibrosis (data not shown) were assessed in hearts of WT male offspring from WT-control and Lepr^db/+^ dams on either the SD or HFD. No differences were found in either parameter among any of the offspring groups.

## Discussion

The adverse maternal environments of GDM and maternal obesity are characterized by maternal leptin resistance and hyperleptinemia [15–19]. As a consequence, there is both reduced leptin signaling in the mother, and exposure of the mother and placenta to high leptin concentrations. Here we evaluated the effect of high maternal leptin, in the absence of maternal hyperglycemia or obesity, on offspring cardiovascular health, with specific emphasis on blood pressure and resistance artery function and structure. There was no difference in blood pressure in offspring of control and Lepr^db/+^ dams, showing that maternal hyperleptinemia is not responsible for the hypertension observed in offspring of diabetic or obese mothers. However, maternal hyperleptinemia significantly impacted mesenteric artery function and structure in offspring, particularly the arterial response to high fat, high sugar diet consumption. These data suggest that maternal leptin interacts in complex ways with other factors in the maternal and postnatal environments to influence vascular health in offspring.

Alterations to resistance artery function and structure have profound effects on the development of hypertension and CVD [33, 37, 38]. Exposure to an adverse maternal environment also can lead to the development of hypertension and CVD [50–52]. However, there is limited information on the role that alterations in resistance artery function and structure play in programming of hypertension by the maternal environment [4–7, 40, 53]. In the offspring of hyperleptinemic dams, differences in resistance artery function were present without hypertension or obesity in the offspring suggesting first, that differences in resistance artery function and structure were directly programmed *in utero*, rather than resulting secondarily from differences in blood pressure or metabolism in the offspring. The absence of significant changes in arterial function or structure in juvenile mice and their presence in adult mice also suggest that the *in utero* effects of maternal hyperleptinemia on the offspring vasculature are mostly programming effects that are expressed only in the mature individual. Moreover, contrary to our initial expectations, maternal hyperleptinemia resulted in beneficial rather than detrimental effects in the offspring vasculature. It increased vasodilatory responses to insulin and increased the passive diameter (outward remodeling) of mesenteric resistance arteries. These beneficial effects, however, occurred only in mice fed a SD. Adverse effects of maternal hyperleptinemia on the offspring vasculature included a specific detrimental response to insulin-induced vasodilation observed only when mice were fed a HFD, and an increase in arterial stiffness that was independent of diet effects.

The observation that changes in arterial function and structure were not associated with significant changes in blood pressure when mice were fed a SD supports the notion that alterations in vascular function and mechanics precede clinical alterations in cardiovascular function [43, 54]. In addition, at least in the SD-fed offspring, programmed alterations in vasomotor responses, vascular remodeling and arterial stiffness may have offset each other, or been offset by other factors that were not measured, like cardiac output or fluid volume, resulting in no net change in blood pressure. Alternatively, it is possible that subtle changes in blood pressure were not detectable by tail cuff because of restraint stress, or by catheter, because of anesthesia.

With regard to vascular function, the observation that there were no significant changes in vascular responses to phenylephrine or SNP suggest that maternal hyperleptinemia had no programing effects on vascular smooth muscle responsiveness to vasoconstrictor or vasodilator agonists. Programing effects of maternal hyperleptinemia on vascular function were particular to the endothelium. Moreover the fact that vessels from SD-fed offspring of Lepr^db/+^ dams had enhanced responses to insulin, but not to ACh, suggest that the beneficial effects of maternal hyperleptinemia on vascular function are associated with improvements in insulin signaling upstream of NO production by endothelial NO synthase (eNOS). This is further supported by the observation that the detrimental effect on vascular function seen in HFD-fed offspring of hyperleptinemic dams consisted of a reduced vasodilatory responsiveness to insulin, but not to ACh. This becomes particularly interesting when one considers that HFD-feeding was associated with an augmented vasodilatory response to ACh in the offspring of WT-control dams, but not in the offspring of hyperleptinemic dams. Enhanced ACh responses following HFD have been shown in offspring of HFD-fed dams before [55] and in obese, diabetic db/db mice, [44] although others have reported reduced ACh response following extended exposure to HFDs [56]. Previous studies have also shown diminished insulin-induced vasodilation following HFD, as occurred in the offspring of hyperleptinemic dams, but only after a longer diet period (10 weeks) [57]. Taken together, these data suggest that maternal hyperleptinemia programs the vascular endothelium in mesenteric resistance vessels not to respond to overnutrition with an enhanced capacity for eNOS-dependent vasodilation and to reduce its responsiveness to insulin. The mechanisms associated with these responses are likely highly complex and remain to be determined.

Alterations in vasomotor responses are often associated with vascular remodeling processes and changes in the physical structure and mechanical properties of the vascular wall [33]. Remodeling is an intricately controlled process that encompasses changes in cytoskeletal organization, cell-to-cell connections and extracellular matrix composition and structure [33–35]. Previously, Souza-Smith et al. [44] showed that over-nutrition in the type 2 diabetic db/db mouse is associated with outward remodeling of the mesenteric resistance circulation. The increase in passive luminal diameter (outward remodeling) was attributed to hemodynamic changes caused by the increased blood flow associated with the characteristic hyperphagia of this animal model. In our current study, mesenteric vessels obtained from offspring of hyperleptinemic dams remodeled outwardly as did those obtained from WT-control dams fed a HFD. It is possible that in the HFD-fed mice outward remodeling was caused by hemodynamic changes attributable to presence of this diet in the gut. However, for the outward remodeling observed in the arteries of offspring from hyperleptinemic dams fed a SD, the only observation that provides a potential mechanism for this phenomenon is the increased vasodilatory responsiveness to insulin seen in the same arteries. The plausibility of this mechanism is supported by the observation that feeding a HFD to offspring of hyperleptinemic dams did not induce outward remodeling in their mesenteric arteries and that this was associated with a reduced vasodilatory response to insulin. As in the study by Souza-Smith et al. [44] outward remodeling of the mesenteric arteries was associated with an increased CSA of the vascular wall, indicating that the remodeling was hypertrophic according to the characterization of remodeling introduced by Mulvany et al. [58].

As in previous studies, HFD consumption increased mean arterial blood pressure, due primarily to an increased diastolic blood pressure [59–61]. However, this increase in blood pressure was observed only in catheter measurements made in anesthetized animals and not in blood pressure measurements obtained using tail-cuff plethysmography. Others have shown that diet-induced obesity increases blood pressure using telemetry [54, 60]. Therefore, it is likely that feeding of a HFD for 8 weeks had started changes in blood pressure regulation that induce hypertension in the present study. However, we cannot discard the possibility that the changes in blood pressure we observed were caused by changes in the sensitivity of the HFD-fed animals to isoflurane.

Mechanically, the arteries from offspring of hyperleptinemic dams had reduced strain levels and were stiffer than arteries from offspring of WT-control dams. This occurred without significant changes in arterial compliance al low pressures. Consumption of a HFD exacerbated the stiffening of arteries in offspring of hyperleptinemic dams, making the elastic modulus of their vessels at low pressures significantly greater than that in vessels from offspring of WT-control dams. This programming effect of maternal hyperleptinemia was not associated with any significant changes in the amount of vascular smooth muscle cells, F-actin stress fibers, elastin or fibrillar collagen contained within the vascular wall. Structurally, the outward hypertrophic remodeling associated with consumption of a HFD was associated with an overall reduction in F-actin and elastin content within the vascular wall. Paradoxically the reduction in elastin content was also associated with a significant reduction in the area occupied by fenestrae in the IEL and a specific reduction in the number of fenestra in the IEL of arteries from offspring fed a HFD that were obtained from hyperleptinemic dams. Consumption of a HFD has been previously shown to be associated with significant reduction in the fenestrae of vessels [43, 62]. Calculation of the elastic modulus normalized as a function of the percolation of the internal elastic lamina and its fenestrae suggests that a reduction in the number and size of fenestrae may participate in augmenting the stiffness of mesenteric arteries in animals fed a HFD [63]. In comparison, the mechanism responsible for the diet-induced vascular hypertrophy in offspring of WT-control dams is not clear, because there were no significant changes in the amount of vascular smooth muscle nuclei, F-actin, fibrillar collagen or elastin contained in the wall of those vessels. It remains to be determined if the change in CSA was caused by presence of bigger cells with less actin stress fibers or by presence of extracellular matrix proteins other than fibrillar collagen. In a previous study of C57Bl/6 fed a 45% fat diet for 32 weeks, outward remodeling of mesenteric arteries occurred in association with adventitial and smooth muscle cell hyperplasia [62].

Further study is needed to untangle the complex underlying mechanisms responsible for functional and structural differences in the vasculature from mice exposed to prenatal maternal hyperleptinemia. Maternal leptin does not cross the placenta to reach the fetal circulation [64, 65]; therefore the observed differences are not due to maternal leptin acting directly on developing fetal vasculature. Rather, maternal hyperleptinemia likely alters maternal metabolism and changes placental function to alter the delivery of nutrients and growth factors to the growing fetus [66–76]. Our other studies indicate that offspring born to hyperleptinemic mothers have better metabolic health overall, with lower body weights, increased spontaneous activity [25], and improved insulin and leptin sensitivity, which may in turn affect vascular function, as exemplified by the increased insulin-dependent vasodilation observed in offspring of hyperleptinemic dams. One possibility is that enhanced leptin sensitivity could affect expression of matrix metalloproteinases [76, 77], which play a key role in artery remodeling [78]. Physical activity is known to slow the progression of CVD and improve vascular homeostasis by decreasing reactive oxygen species and increasing NO bioavailability in the endothelium [79].

Overall, this study indicates that exposure to high leptin levels *in-utero* affects vascular function in a manner dependent on the vasoactive stimulus and postnatal diet. Maternal hyperleptinemia was largely beneficial to vascular function when offspring were fed a SD, and deleterious when they were fed a HFD. This is supportive of the hypothesis that alterations in maternal serum leptin may contribute to the changes in cardiovascular health observed in offspring of obese or diabetic pregnancies. This study also strengthens the idea that programming of arterial function may precede changes in blood pressure, and thus, may be a key mechanism by which maternal environment can alter cardiovascular health [40, 53]. While alterations to vascular function are linked to the development of hypertension, it is important to note that these changes appear to occur prior to the onset of hypertension and that multiple vascular changes are observed in multiple vascular beds. Thus, studies over a longer time course, and in other vascular beds, may be necessary to fully understand whether vascular changes induced by maternal hyperleptinemia persist and lead to overt beneficial or adverse changes in blood pressure and CVD.
